# Quadratic variance models for adaptively preprocessing SELDI-TOF mass spectrometry data

**DOI:** 10.1186/1471-2105-11-512

**Published:** 2010-10-13

**Authors:** Vincent A Emanuele, Brian M Gurbaxani

**Affiliations:** 1 School of Electrical and Computer Engineering, Georgia Institute of Technology, Atlanta, GA, USA; 2 Chronic Viral Diseases Branch, Division of High-Consequence Pathogens and Pathology, National Center for Emerging and Zoonotic Infectious Diseases, Centers for Disease Control and Prevention, Atlanta, GA, USA

## Abstract

**Background:**

Surface enhanced laser desorption/ionization time-of-flight mass spectrometry (SELDI) is a proteomics tool for biomarker discovery and other high throughput applications. Previous studies have identified various areas for improvement in preprocessing algorithms used for protein peak detection. Bottom-up approaches to preprocessing that emphasize modeling SELDI data acquisition are promising avenues of research to find the needed improvements in reproducibility.

**Results:**

We studied the properties of the SELDI detector intensity response to matrix only runs. The intensity fluctuations and noise observed can be characterized by a natural exponential family with quadratic variance function (NEF-QVF) class of distributions. These include as special cases many common distributions arising in practice (e.g.- normal, Poisson). Taking this model into account, we present a modified Antoniadis-Sapatinas wavelet denoising algorithm as the core of our preprocessing program, implemented in MATLAB. The proposed preprocessing approach shows superior peak detection sensitivity compared to MassSpecWavelet for false discovery rate (FDR) values less than 25%.

**Conclusions:**

The NEF-QVF detector model requires that certain parameters be measured from matrix only spectra, leaving implications for new experiment design at the trade-off of slightly increased cost. These additional measurements allow our preprocessing program to adapt to changing noise characteristics arising from intralaboratory and across-laboratory factors. With further development, this approach may lead to improved peak prediction reproducibility and nearly automated, high throughput preprocessing of SELDI data.

## Background

Mass spectrometry is a promising technology for biomarker discovery [1]. There are a wide variety of mass spectrometers from which one could choose from during the design of a biomarker discovery experiment, reviewed in [2]. Matrix assisted laser desorption/ionization time-of-flight mass spectrometry (MALDI-TOF MS, or just MALDI) can ionize whole proteins intact over a wide range of protein mass values, making it suitable for biomarker discovery in complex media such as blood serum, where both protein concentrations and masses vary greatly [3]. Surface-enhanced laser desorption/ionization time-of-flight mass spectrometry (SELDI-TOF MS, or just SELDI) [4] is a variant of MALDI that adds an on-chip chromatographic separation step at the front end of the analysis pipeline. This, combined with robot-automated sample preparation, enables SELDI to be high-throughput, an attractive feature for many laboratories. For a recent review of the application of SELDI in the context of biomarker discovery, see [5].

The typical SELDI work flow involves the collection of samples (e.g.- blood serum) from patients, application of the samples to SELDI ProteinChips^® ^selected for desired physicochemical properties, and analysis in the SELDI mass spectrometer. The raw data must be preprocessed to detect relevant peaks which correspond to proteins in the sample. Typical signal preprocessing steps performed are spectral alignment, denoising/smoothing, peak detection, peak matching, normalization, and quantification (see Figure 1 of [6]). The preprocessing of the raw SELDI spectra is typically accomplished using one of several available software packages (reviewed in [6-8]). Artifacts due to insufficient preprocessing of the data have, in the worst case, led to erroneous biological conclusions in early SELDI studies [9-11]. This fact inspired several important comparison studies of SELDI preprocessing algorithms [6-8,12]. We now briefly summarize a few of the major contributions. For a more detailed overview, see the introduction of [6].

Coombes *et al *introduced the use of wavelets for denoising SELDI spectra [13], providing a more adaptive approach to denoise compared to moving average filters (e.g., as in [14]). Meanwhile, Morris *et al *introduced the notion of a mean spectrum, which represents average protein activity of a group of spectra. Under non-restrictive assumptions, the mean spectrum has less noise and allows one to circumvent complicated peak matching algorithms that consolidate peak predictions among individual spectra into a consensus prediction. Malyarenko *et al *introduced a novel baseline removal algorithm based on a proposed charge accumulation model of the saturation phenomenon of the detector [15]. This was one of the first algorithms that was designed from the "bottom-up", starting with physical considerations of SELDI. Later, deconvolution filters were shown to be a possible approach for improving mass resolution of SELDI [16-18].

Sköld *et al *analyzed single-shot spectra [19], the basic components of a final SELDI spectrum obtained by summing the results of many laser shots. They suggested that the observed counts in the single shot spectra may be proportional to a Poisson random variable, proposing a heteroscedastic model for the data. Meuleman *et al *also make use of single-shot spectra (sub-spectra) to derive a preprocessing algorithm based on analyzing these components separately [20].

In an attempt to improve on the bottom-up approach to preprocessing, we analyze the statistics of the SELDI signal over a wide range of intensity values. Based on data presented herein, we propose a natural exponential family model with quadratic variance function for the statistics of the detector response for SELDI experiments. We believe this model is a plausible explanation for acquisition of single-shot spectra, summing of single-shot spectra into a final spectrum, and extracting protein estimates from a mean spectrum under a unified framework. Under this framework, we introduce a new preprocessing approach, adaptive to changing noise characteristics per spectrum and per experiment, and show favorable peak prediction performance.

## Results

### Buffer-only intensity measurements

Electronic measurements exhibit natural random fluctuations [21]. In many cases, these fluctuations are independent of the signal and are modeled as additive white Gaussian noise. In order to understand the nature of the noise fluctuations inherent to SELDI, we study the response of the detector under controlled experiments applying different buffers instead of protein samples under varying laser intensities (as in [22]). This eliminates the complexity introduced by adding serum to the chips while facilitating measurements of ion counts over a wide range of intensity values. In principle, this gives us a set of *n *repeated experiments from which we can study the statistics of the detector response compounded with noise and interference inherent to SELDI. In this fashion, we have generated two separate buffer + matrix datasets, denoted BUFFER1 and BUFFER2, which represent data generated on the *same *SELDI PBS IIc machine by *different *scientists and different machine parameters. BUFFER1/BUFFER2 contain 183/114 spectra, respectively.

**Figure 1:**
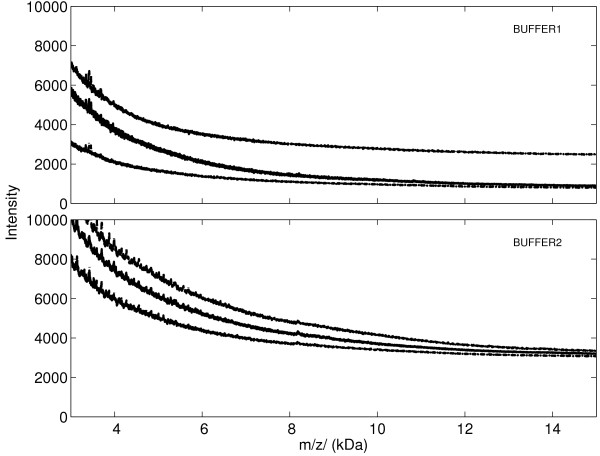
**Quantile spectrum visualization of BUFFER1 and BUFFER2 datasets**. Quantile spectrum visualizations for all 183/114 spectra from BUFFER1/BUFFER2 datasets respectively. The middle, upper, and lower spectra are the 50% (median), 75%, and 25% quantile spectra respectively, calculated pointwise for each mass point. The results show that different machine settings give rise to different statistical behavior of the intensity values registered at the detector. Preprocessing techniques should be able to adapt to this varying behavior.

We visualize all of the spectra in BUFFER1 and BUFFER2 in Figure 1. In particular, we are interested in analyzing the region between 3 and 30 kDa, since this is the mass focusing region in our experiments. In this region, the observations across spectra for a fixed time (mass) point represent approximately independent, identically-distributed measurements in BUFFER1 or BUFFER2, respectively. Figure 1 shows the median, 75% quantile, and 25% quantile of BUFFER1 and BUFFER2. The median spectrum shows the form of an ordinary measurement, with any measurement between the 75% and 25% spectrum lines considered typical as well.

Figure 1 shows us the behavior of the typical buffer + baseline signal component seen in all SELDI raw spectra. Indeed, we see that changing different machine settings leads to different response properties. For BUFFER2, the median spectral response is large in the range shown, and the distribution of responses is symmetric about the median, whereas the distribution of detector response values for BUFFER1 are heavily skewed, and thus certainly not normally distributed.

**Figure 2:**
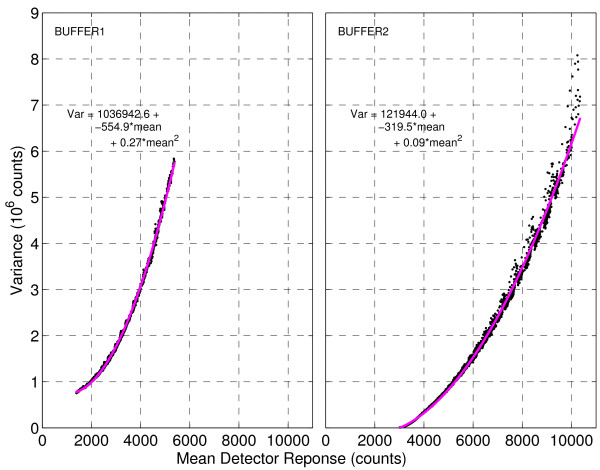
**SELDI detector intensity response curves**. For repeated experiments under homogeneous machine settings, the variance in intensities observed is shown to be quadratic in the mean intensity observed. Thus, peaks occurring in areas of the spectrum affected near the baseline will be more noisy and more difficult to detect. Most algorithms for preprocessing SELDI data assume constant variance, independent of signal intensity. The detector response curve is shown to be dependent on machine settings, as it is different for BUFFER1 and BUFFER2.

We study the detector response (intensity output) for SELDI under varying input conditions, creating a detector response curve as follows. For each fixed time (mass) point across spectra from BUFFER1 in the mass focused region [3*kDa*; 30*kDa*], we estimate the mean intensity observed and the corresponding variance, with the same repeated for BUFFER2. These are displayed as a scatter plot in Figure 2 along with the best fit quadratic curve. Observing Figure 2 we see

1. Intensity fluctuation/variance increases monotonically with the mean.

2. The variance of the detector response is a quadratic function of the mean, to a very good approximation

3. The detector response curves for BUFFER1 and BUFFER2 are quite different, and thus are dependent on the machine settings.

The detector response statistics thus exhibit a quadratic variance function. Briefly, a random variable *X *is said to have a quadratic variance function (QVF) if

(1) $$V\left(\mu \right)=\upsilon_{0}+\upsilon_{1}\mu +\upsilon_{2}\mu^{2},$$

with *μ *being the mean of *X*, *V*(*μ*) the variance, and *υ*_*0*_, *υ*_1_, *υ*_2 _constants, some of which may be zero.

From these observations, summarized in Figures 1 and 2, it seems unlikely that an algorithm optimized for BUFFER1 would work well on BUFFER2 and vice versa. Further, neither a homoscedastic approach (e.g. - standard wavelet shrinkage [23]) or a simple heteroscedastic approach (e.g. - Poisson regression formulation [24]) to preprocessing the data is likely to be sufficient.

### Data for evaluating preprocessing algorithms

We have generated two new datasets for evaluating preprocessing algorithms in order to improve upon purely simulation-based datasets used in previous comparison studies [6,7]. A good comparison dataset should have the following properties (discussed previously in [6]):

1. Exact protein content is known (and thus expectation of where "true" peaks will appear)

2. Analyzed sample is complex containing many proteins/peaks

3. Noise and baseline characteristics should be as close to those of real SELDI data as possible.

If one uses simulated data [6,7,25], complete control can be attained over requirements 1) and 2) at the expense of having noise/baseline characteristics that are overly ideal. If one uses purely real data, the noise, baseline, and artifacts that arise in actual experiments are present. However, this usually accompanies the trade-off of either not knowing the exact protein content (e.g.- complex serum data) or an overly simplified scenario (e.g. - spike-in data).

**Figure 3:**
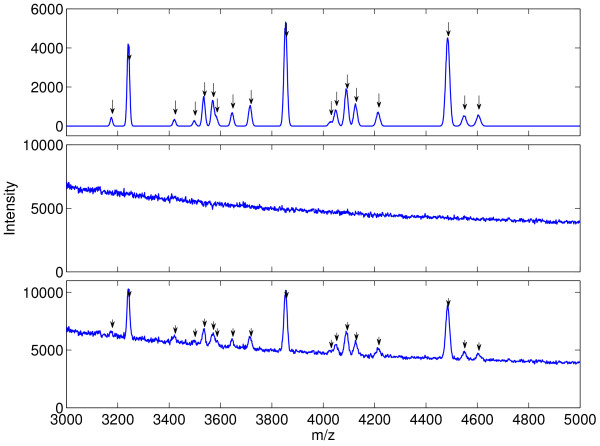
**Construction of hybrid simulated/real spectra for testing preprocessing programs**. (top) Clean, pure protein component spectrum with no noise and no baseline simulated using SimSpec 2.1 MALDI/SELDI simulation engine. Arrows over peaks show the *m/z *values of the virtual proteins. (middle) Buffer+matrix spectrum generated in a SELDI PBS IIc, representing noise, baseline, and artifacts that are typically seen. (bottom) Final hybrid spectrum, consisting of the sum of simulated and real components. Hybrid spectra have the advantage of having diverse signal components (150 virtual proteins) with *exact *knowledge of the virtual proteins while retaining the true noise and baseline characteristics from real SELDI data.

We combine the advantages of purely simulated and real data by introducing the notion of a hybrid spectrum. To generate a hybrid spectrum, we use an implementation of the SimSpec 2.1 SELDI simulator [25,26]http://bioinformatics.mdanderson.org/Software/Cromwell/simspec.zip to generate a "clean" SELDI spectrum, shown at the top of Figure 3. This gives an accurate peak shape characteristic as would be seen in low resolution SELDI/MALDI for given mass and ion abundance values, without any electronic noise or baseline present. We then select one of our buffer + matrix spectra (from either BUFFER1 or BUFFER2) and add the two together to produce the hybrid spectrum shown at the bottom of Figure 3. Thus, in a hybrid spectrum we know the *exact *virtual protein content specified to the simulator a *priori *while maintaining *exactly *the same noise, baseline, and other artifacts one encounters with real SELDI data.

Further details on the hybrid spectra can be found in the Methods section and in Additional file 1. The collection of hybrid spectra under different operating conditions results in test sets, denoted HYBRID1 and HYBRID2, with each test set containing thirty datasets of fifty hybrid spectra each. The mean performance of a preprocessing algorithm on HYBRID1 and HYBRID2 can be interpreted as the expected performance of the preprocessing approach in each separate operating condition in a repeated experiment or sampling from a homogeneous population (e.g. - cancer group or control group).

### New preprocessing algorithms for SELDI

We have developed a set of MATLAB^® ^scripts for preprocessing SELDI spectra named LibSELDI. For information on how to obtain LibSELDI and the associated scripts used to produce the figures in this paper, contact the authors. We compare our preprocessing package to the MassSpecWavelet package from the Bioconductor project [27]. MassSpecWavelet has been established as one of the best approaches in terms of peak finding in recent comparison studies [6,7], and has been downloaded > 6000 times in the past two years as of March 2010 http://bioconductor.org/packages/stats/bioc/MassSpecWavelet.html. Both packages have the advantage of having only one main user-adjusted parameter.

**Figure 4:**
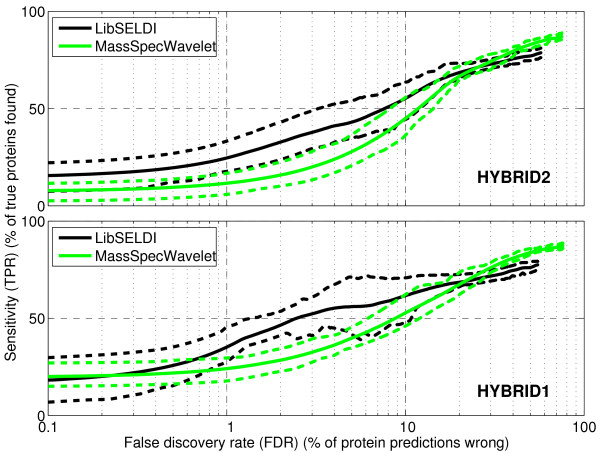
**Trade off between sensitivity and false discovery rate for LibSELDI and MassSpecWavelet**. Average loess-smoothed operating characteristics show the trade-offs between sensitivity (TPR) and false discovery rate (FDR) for HYBRID1 and HYBRID2. The mean loess-smoothed curve is indicated by the solid line, while the upper and lower dashed lines indicate the 75% and 25% quartile curves. The FDR axis is shown in log-scale to emphasize lower FDR values. LibSELDI demonstrates superior sensitivity compared to MassSpecWavelet on both datasets for FDR values less than about 25%. MassSpecWavelet has the advantage for FDR values greater than 25%.

In order to compare the performance of each preprocessing program, we generate operating characteristic curves (OC curves) [6,20], one for each of the 30 datasets of HYBRID1 and HYBRID2, by varying the Peak Area threshold (LibSELDI) and signal-to-noise ratio threshold (Snr.Th in MassSpecWavelet) parameters in the programs. Code snippets showing how MassSpecWavelet was tested can be found in Additional file 1. This allows us to understand the trade-offs between false discovery rate (FDR) and sensitivity (TPR) achieved by each algorithm. The results for both the HYBRID1 and HYBRID2 collections are shown in Figure 4, where we have plotted the FDR-axis in log scale to emphasize the low FDR region which is usually of most interest in biomarker discovery applications. Note that, since both HYBRID1 and HYBRID2 are collections of datasets representing repeated trials (or equivalently a homogeneous population), the OC curves we show in Figure 4 are the mean OC curves across the 30 datasets for each.

The results show that LibSELDI tends to have a considerable advantage in the low FDR region, while MassSpecWavelet tends to have higher sensitivity for *FDR *> 25%. One way to summarize the performance of the algorithms is using the area under the OC curve for the FDR region of interest. We compute two area under the curve values, PAUC [6] (calculated for *FDR *∈ [0, 50%]), and PAUC25 (calculated for *FDR *∈ [0, 25%]). The results are shown in Table 1, where we have normalized each score separately so that a perfect PAUC25 (likewise, PAUC50) score is 100.

**Table 1:** Area under the operating characteristic comparison

Algorithm/Dataset	PAUC25	PAUC
LibSELDI/HYBRID1	58.8% (9.9)	66.1% (7.1)

MassSpecWavelet/HYBRID1	50.8% (8.5)	64.9% (5.9)

LibSELDI/HYBRID2	53.2% (8.7)	64.1% (6.1)

MassSpecWavelet/HYBRID2	45.4% (9.5)	61.3% (6.9)

Area under the operating characteristic curve in a range of false discovery rate values of interest is a useful way to compare peak prediction performance. We show two partial area under the curve metrics, calculated in the range *FDR *∈ [0, 50%] (PAUC) and *FDR *∈ [0, 25%] (PAUC25). PAUC is more of overall measure of peak prediction potential, while PAUC25 focuses on measuring performance at low FDR. The number shown is the average (standard error) calculated from the 50 operating curves from HYBRID1 and HYBRID2. LibSELDI shows particularly appealing PAUC25 performance.

**Figure 5:**
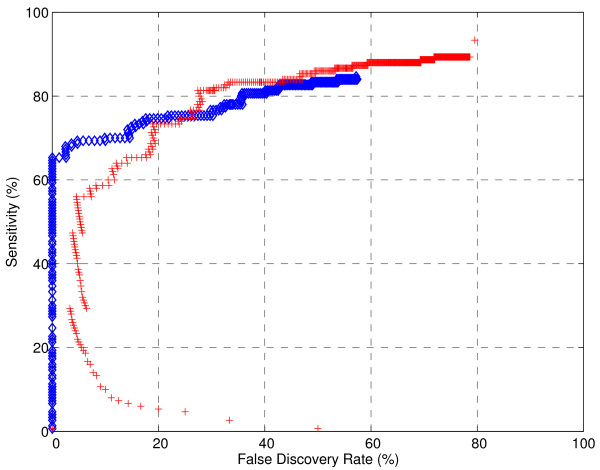
**Example operating characteristic**. Operating points shown summarize the performance of LibSELDI and MassSpecWavelet on Dataset 2 of HYBRID1 for many different parameter choices. Each blue diamond is the (FDR, TPR) observed for a single choice of Peak Area threshold for LibSELDI, while each red plus symbol shows the result of a single Snr.Th parameter choice for MassSpecWavelet. For this particular example, LibSELDI finds more than 90 true proteins before making a mistake. At high FDR conditions, MassSpecWavelet resolves close to 90% of proteins compared to about 85% for LibSELDI.

In Figure 5, we show the specific operating characteristics for LibSELDI and MassSpecWavelet for Dataset 2 of HYBRID1. While both algorithms perform well, LibSELDI resolves more than 90 proteins correctly before making a mistake. Since operating characteristics show false discovery rate along the x-axis rather than false positive rate (as in the traditional ROC curves), they tend to penalize more when false predictions are made with very few true proteins found. Indeed, in this case MassSpecWavelet got its first protein prediction correct but its second prediction wrong, leading to the point at FDR = 50%, TPR = 7%. Thus, operating characteristics with false discovery rate along the x-axis enforce the principle of conservative decision making, rewarding approaches that are successful with their initial large threshold (conservative) predictions and penalizing those that make mistakes early.

At FDR values greater than 30%, MassSpecWavelet outperforms LibSELDI. However, this is at the expense of generally more promiscuous predictions, since MassSpecWavelet generates 586 potential protein predictions compared to 250 for LibSELDI.

## Discussion

We posit that the detector response is a member of the Natural Exponential Family with Quadratic Variance Function (NEF-QVF), which is a proper subset of the exponential family of distributions [28]. Figures 1 and 2 show that assuming the detector response takes the form of a specific distribution is impractical, but that the detector response *V*(*μ*) has a QVF. The NEF-QVF family of distributions occur often in practice and have the following useful properties, characterized by Morris [28]:

1. If a random variable *X *∈ NEF-QVF, it is completely specified by its variance function *V*(*μ*)

2. If *X *∈ NEF-QVF, *a*, *b *constants then *aX *+ *b *is also NEF-QVF

3. **Additivity**: If *X*_1_; *X*_2 _**∈ **NEF-QVF, then *X*_1 _+ *X*_2 _is NEF-QVF

4. Affine combinations of normal, Poisson, gamma, binomial, negative binomial, and generalized hyperbolic secant distributed random variables generate all possible distributions in the NEF-QVF family.

There are some physical reasons as to why the NEF-QVF assumption could be reasonable as well. Some plausible justifications for the first two terms in Eq. (1) are:

1. **Constant Term**: This is possibly due to thermal noise (additive Gaussian noise) which is common to all electronic measurement devices [21]

2. **Linear Term**: The ability to detect an ion in a multiple stage electron multiplier, a common type of detector in MALDI-like instruments, is described by compound Poisson statistics [29].

The existence of a plausible physical explanation for the quadratic variance term remains an open question. However its effect is measured in both BUFFER1 and BUFFER2 and cannot be neglected. While the QVF model explains the data well in the mass focused region between 3 and 30 kDa, it is likely to break down at lower masses around 2-2.5 kDA where the baseline reaches a maximum. In this region the detector often saturates, introducing a non-linearity into the data that we have not accounted for.

The success of our univariate model for SELDI may indicate that we have selected the most important feature to consider in the preprocessing of the data: namely, the fluctuations in the response of the ion detector subject to different inputs. The analysis of expression values of preprocessed data, on the other hand, requires multivariate methods as there are significant statistical dependencies between the peak heights corresponding to proteins that may be interacting. While these correlations are important in the analysis performed after the data is preprocessed, our results indicated it may be safe to ignore them during the preprocessing. While we have shown LibSELDI to be accurate for estimating peak m/z values, we have not assessed the usefulness approach for estimating peak intensities in this work. The utility of LibSELDI for accurately estimating peak intensities remains an open question and subject of future work.

It is entirely possible that the quadratic variance model could be applicable to other similar technologies such as MALDI and newer SELDI mass spectrometers. This, however, has not been confirmed.

Having buffer only spectra allows one to estimate the parameters of the detector response curve. Knowledge of the detector response curve enables us to apply the modified Antoniadis-Sapatinas denoising scheme described in the methods. Using this approach in our LibSELDI package yields excellent peak detection performance. We have proved this concept on HYBRID1 and HYBRID2 by estimating the QVF parameters of (1) using the buffer-only spectra that were randomly selected from BUFFER1 and BUFFER2 respectively. This implies that spots on SELDI chips should be reserved for buffer-only spectra. Thus, the trade-off for using our approach is increased cost in terms of the number of chips one must use. The modified Antoniadis-Sapatinas denoising is computationally intensive as well, taking approximately seven minutes per spectrum on a high-end workstation.

We argue that some of the cost is recovered by the potential for adaptive and accurate preprocessing, but not all. It may be possible to use QC and/or calibration samples to estimate the QVF as well rather than buffer-only spots. However, this would add in some additional variation due to the nature of the medium (serum, plasma, etc).

While LibSELDI outperforms MassSpecWavelet on the HYBRID1 and HYBRID2 test sets, the applicability of this comparison and of these results to purely real data remains an open question. There is some basic biological variability modeled in our test sets (see description in supplement of [6]). However, data from complex biological samples such as serum or plasma likely contains more biological variation and artifacts than we have modeled in HYBRID1 and HYBRID2. The investigation of how biological variation affects the model in QC samples is a work in progress.

**Figure 6:**
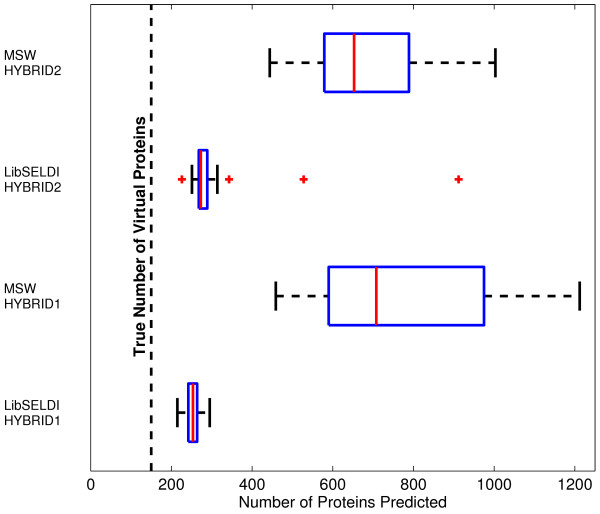
**Efficiency of peak/protein predictions**. We show boxplots summarizing the number of peaks predicted for each program in the mean spectrum of each dataset from HYBRID1 and HYBRID2 before thresholding. LibSELDI consistently predicts around 250 peaks, while MassSpecWavelet predicts more than 600 peaks consistently. MassSpecWavelet's more promiscuous predictions lead to high sensitivity at the expensive of higher false discovery rate performance. LibSELDI's peak predictions are reproducibly closer to the true number of virtual proteins, 150 of them, present in each dataset.

In addition to achieving a better mean OC curve at lower FDR values, LibSELDI consistently predicts fewer peaks than MassSpecWavelet, leading to protein predictions closer to the true number of proteins in the data, as shown in Figure 6. This is further evidence that the adaptive modified Antoniadis-Sapatinas denoising approach using the NEF-QVF model for the detector response is smoothing the spectra by close to the right amount.

## Conclusions

We have shown that the variance of the intensity of a SELDI spectrum is quadratic in the mean signal strength. We further make the flexible assumption that the underlying distribution of the intensities is from a natural exponential family. From this point of view, we use a modified Antoniadis-Sapatinas wavelet shrinkage approach for denoising SELDI spectra. With this method at the core of our LibSELDI program for preprocessing SELDI data, we demonstrate excellent sensitivity at low false discovery rates. For applications that can tolerate higher false discovery rates, the MassSpecWavelet algorithm performs better in this region.

Our work has implications in the design of SELDI experiments. Namely, the modified Antoniadis-Sapatinas denoising technique performs well but requires an estimate of the quadratic variance function (QVF) describing the SELDI detector. This, in turn, is affected by machine settings. We have used buffer-only spectra to estimate the QVF. Thus, buffer-only spots could be interlaced on chips. We are investigating less expensive ways to estimate the QVF in future work.

## Methods

### Protocol for generating buffer-only spectra

Buffer-only spectra were generated by interspersing buffer only samples with protein samples from subjects (e.g. serum samples) and with pooled subject samples (for quality control) on the same chip. The buffer-only samples were spotted with wash buffer that was either PBS (phosphate buffered saline with various concentrations of phosphate and NaCl) based or acetonitrile + TFA (triflouroacetic acid) based, as manufacturer recommended per chip type. These buffer only samples were processed with the same washing steps as the subject samples, as described in [22], and then SPA matrix was applied to all spots.

The samples were analyzed with the Protein Biological System IIc™ SELDI mass spectrometer (Ciphergen Biosystems, Freemont, CA). The machine settings (e.g. laser intensity, detector sensitivity) and precise washing steps varied from buffer only spot to buffer only spot, and were generally different between BUFFER1 and BUFFER2. Note especially that laser intensities were generally higher for BUFFER2 than for BUFFER1. A detailed list of machine settings is given in the Additional file 1.

### Hybrid data

Calculating performance statistics for comparison of MassSpecWavelet and LibSELDI requires a large number of spectra emulating an experiment that was repeated many times. To generate the HYBRID1 dataset, we combine each clean spectrum with one buffer+matrix spectrum from BUFFER1, and similarly we form HYBRID2 from BUFFER2 by combining those spectra with the same clean spectra.

A basic model of repetitive experiments for SELDI is available with SimSpec 2.1 that takes into account fluctuations in protein concentrations, m/z values, and prevalence in the data. Using the SimSpec 2.1 model developed at the MD Anderson Cancer Center [25,26], we generate 30 datasets containing 50 clean (noise and matrix-free) spectra each. Each dataset consists of 150 virtual proteins and each spectrum within the given dataset contains a proper subset of these proteins with fluctuating parameters according to the model described in [25] and its supplement. The goal for the preprocessing programs in our performance evaluation is to reconstruct the master list of 150 virtual proteins characterizing the dataset. Repeated across all 30 datasets, we can calculate useful performance statistics. The properties of the 150 virtual proteins themselves are drawn from a prior distribution that was estimated from real data. See [25], or alternatively, the description in the supplement of [6].

We use sampling to overcome the limitation of having much fewer spectra in BUFFER1 and BUFFER2 than we have clean spectra in preparation for testing the algorithms. In principle the best way to construct the hybrid test sets would be to have one unique spectrum in BUFFER1 (likewise BUFFER2) for each spectrum in our clean protein-only set. However, this would require 1500 buffer+matrix runs to be performed for both BUFFER1 and BUFFER2, an impractical amount of blank chips to run. Sampling from BUFFER1 (BUFFER2) provides a cost effective way to introduce variation in the noise/matrix characteristics between the datasets in HYBRID1 (HYBRID2).

### Preprocessing the spectra

First we consider a model for a single SELDI spectrum, *X*(*t*). We observe *X*(*t*), a random process, on a discrete time grid *t*_1_,..., *t*_*m*_, where *X*(*t*) represents the intensity of the raw SELDI spectrum observed at time (equivalently mass) point *t*. For all *t*, we assume that *X*(*t*) is distributed according to a natural exponential family (NEF) with quadratic variance function (QVF) equal to *V*(*μ*(*t*)) as in Eq. (1). The variance function *V*(*μ*) completely characterizes the NEF-QVF family. The goal of preprocessing in SELDI is to estimate *μ*(*t*), the expectation of *X*(*t*), which is the signal corresponding to ions that hit the detector. With a good estimate of *μ*(*t*), extracting peaks and estimating protein *m*/*z *values in a dataset is relatively straightforward.

As a side note we point out that a SELDI spectrum is actually a sum of single shot spectra. However, the additivity property of the NEF-QVF family guarantees the sum is NEF-QVF provided that the single-shot spectra are NEF-QVF, agreeing with our detector response model and experimental observations.

#### Multiple spectra considerations

Rather than observe a single spectrum, the typical biomarker discovery approach is to generate at least one spectrum for each of *n *samples from an approximately homogeneous population. For example, one homogeneous population may be a group of early stage prostate cancer patients matched for age, race, etc. Assuming the samples are run on the same SELDI machine with the same operating conditions, we have

(2) $$X_{1}\left(t\right),...,X_{n}\left(t\right)\propto \;\text{NEF-QVF}\left(V\left(\mu \left(t\right)\right)\right).$$

Our assumption that all *n *patients have the same underlying *μ*(*t*) is equivalent to assuming that the underlying biological condition being observed in each patient is approximately the same. Thus, we wish to estimate the underlying commonality *μ*(*t*) related to the biology of their condition expressed through the SELDI signal. We can mitigate some of the effects of the QVF by forming the mean spectrum (first introduced by [25]).

(3) $$X_{•}\left(t\right)=\frac{1}{n}\sum_{k=1}^{n}X_{k}\left(t\right).$$

It is straightforward to show that

(4) $$E\left\{X_{•}\left(t\right)\right\}=\mu \left(t\right)$$

(5) $$\text{Var}\left(X_{•}\left(t\right)\right)=\frac{1}{n}V\left(\mu \left(t\right)\right).$$

Thus, the mean spectrum concept is valuable under the assumptions of the NEF-QVF model as well.

#### Modified Antoniadis-Sapatinas denoising

We now discuss estimation of *μ*(*t*) from the mean spectrum (3). Since the *X*_*k*_(*t*) are sampled on a discrete time grid (and thus *X*_•_), we introduce vector notation

$$\begin{array}{c} x_{•}={\left[X_{•}\left(t_{1}\right),...,X_{•}\left(t_{m}\right)\right]}^{\prime }\\ \mu ={\left[\mu \left(t_{1}\right),...,\mu \left(t_{m}\right)\right]}^{\prime }. \end{array}$$

For any estimate $\hat{\mu }\left(x_{•}\right)$ of, ***μ ***we measure its fitness using the mean-squared-error (MSE)

(6) $$M\;S\;E\left(\hat{\mu }\left(x_{•}\right),\mu \right)=E\left\{{\left\|\hat{\mu }\left(x_{•}\right)-\mu \right\|}^{2}\right\}.$$

Antoniadis and Sapatinas proposed a wavelet shrinkage scheme to solve for $\hat{\mu }$ in (6) in the context of NEF-QVF regression [30]. We summarize their main results. For our denoising, we use the orthogonal discrete wavelet transform with respect to the Symmlet 8 basis [31]. The transform can be represented by an *m *× *m *orthogonal matrix W,

(7) $$w=Wx_{•}\text{.}$$

Let **h **be a length *m *vector with entries taking values between 0 and 1. Let *H *= diag(**h**) be the *m *× *m *matrix defined by placing the entries of **h **along the main diagonal, all other entries 0. The class of estimators for $\hat{\mu }\left(x_{•}\right)$ considered by [30] take the form

(8) $$\begin{array}{c} \hat{\mu }\left(x_{•}\right)=W^{\prime }Hw\\ =W^{\prime }HWx_{•}. \end{array}$$

This is the typical wavelet denoising scenario where each wavelet coefficient is left alone or shrunk towards zero according to some criterion, and is completely defined by the vector **h**. Antoniadis and Sapatinas showed that a good estimator for data from the NEF-QVF family is given by choosing

$$\begin{array}{l} \begin{array}{cc} \tilde{h}\left(i\right)=\frac{\left[w{\left(i\right)}^{2}-{\hat{\sigma }}^{2}\left(i\right)\right]+}{w{\left(i\right)}^{2}}, & i=1,...,m \end{array}\\ \left[z\right]+=\{\begin{array}{cc} z, & z\geq 0\\ 0, & z<0. \end{array} \end{array}$$

The term ${\hat{\sigma }}^{2}$ is estimated as

(9) $${\hat{\sigma }}^{2}=\frac{1}{1+\upsilon_{2}}\left(W\cdot W\right)V\left(x_{•}\right).$$

Where *V*(**x**_**•**_) is the vector constructed by applying the QVF from (1) to each term of **x**_**•**_. (*W *· *W *) is the matrix whose *i, j *element is the square of the *i*, *j*element of *W*. The parameters *υ*_0_, *υ*_1_, *υ*_2 _in (1) are measured from the buffer-only spectra, as described in the Results and Discussion section.

We make an intuitive modification to (9)

$$\begin{array}{c} {\tilde{\sigma }}^{2}=\frac{1}{1+\upsilon_{2}}\left(W\cdot W\right)V^{†}\left(x_{•}\right).\\ V^{†}\left(x_{•}\left(i\right)\right)=\max\left\{V\left(x_{•}\left(i\right)\right),\upsilon_{0}\right\}. \end{array}$$

Thus our modified Antoniadis and Sapatinas estimator $\tilde{h}$ uses ${\hat{\sigma }}^{2}$ in (8) rather than ${\hat{\sigma }}^{2}$. The modification was introduced to account for cases when (9) may underestimate the noise when low amounts of observed signal are detected. Define

$$\begin{array}{l} \tilde{h}=\frac{\left[w{\left(i\right)}^{2}-{\tilde{\sigma }}^{2}\left(i\right)\right]+}{w{\left(i\right)}^{2}}\\ \hat{H}=\text{diag}\left(\tilde{h}\right). \end{array}$$

Then, our modified Antoniadis-Sapatinas estimate of ***μ ***is defined as

(10) $$\tilde{\mu }=W^{\prime }\tilde{H}Wx_{•}.$$

#### Peak detection/baseline removal

We consolidate the two preprocessing steps of baseline removal and peak detection typically performed separately into a single step as follows. We assume that the underlying *μ*(*t*) shown in (4) is the superposition of protein ions, *s*(*t*), and energy-absorbing matrix ions, *b*(*t*) striking the detector. It is well known that the distribution of the isotopes in our analyte of interest gives rise to a roughly Gaussian peak shape. Thus, we propose

(11) μ(t)=s(t)+b(t)

(12) $$s\left(t\right)=\sum_{j}a_{j}G_{3}{{}_{\sigma }}_{{}_{j}}\left(t_{j},\sigma_{j}\right)$$

where, $G_{\alpha }(t_{j},\sigma_{j})$ denotes a Gaussian kernel function centered at *t*_*j *_with standard deviation *σ*_*j *_and zero outside the interval [*t*_*j *_- *α*, *t*_*j *_+ *α*].

Typically, *s*(*t*) is very sparse in the sense that it is mostly zero over the domain of the observed signal. Therefore, the local minima of our estimated baseline + noise signal $\tilde{\mu }$ are points we may assume touch the baseline. From this point of view, once we have detected all the local minima in $\tilde{\mu }$, the baseline curve estimation problem reduces to an interpolation problem amongst these points. We have found through experimentation that piecewise cubic Hermite interpolating polynomials [32] are excellent interpolation functions.

The minima and maxima in $\tilde{\mu }$ are found in one pass using the extrema function downloadable from MATLAB^® ^central file exchange. The maxima are the peaks in the mean spectrum potentially indicating proteins represented in our sample population while the minima correspond to samples from the baseline signal.

Each detected peak is quantified using peak area and a threshold is chosen based on the peak area measurement to generate the final prediction set.

### Operating characteristics

The peaks we detect in $\tilde{\mu }$ represent the initial set from which we choose our final estimates of proteins that are active in the population of interest. The choice of final estimate is accomplished using a peak area threshold (LibSELDI) or signal-to-noise ratio measurement (Snr.Th in MassSpecWavelet). From each prediction, we calculate the observed false discovery rate (FDR) and true positive rate (TPR, also called sensitivity)

(13) $$FDR=\frac{FP}{FP+TP}$$

(14) $$TPR=\frac{TP}{TP+FN}.$$

Where TP (the number of true positives) is the number of the 150 virtual protein *m*/*z *values having at least one predicted *m*/*z *value within 0.3% relative error. The FP is defined as the number of predicted *m*/*z *values not within 0.3% of any of the 150 virtual protein *m/z *values for this dataset. Similarly, FN is the number of the 150 virtual protein values without any predicted *m*/*z *value within 0.3% relative error.

For each dataset, a curve is fit to the operating points. Each operating curve is averaged to produce a mean operating characteristic, as shown in Figure 4. From this curve, the calculation of the area-under-the curve is straightforward. For more details, see sections 2.2 and 2.2.1 of [6].

## Authors' contributions

VE and BG conceived and developed the theoretical aspects of the study, analyzed the results, and wrote the manuscript. VE developed LibSELDI and ran the simulations. Both authors have read and approve of the manuscript.

## Supplementary Material

Additional file 1 **Experiment and simulation settings**. This file contains additional details about how simulations and experiments were carried out. Click here for file
