# Supplementary material for: Quadratic variance models for adaptively preprocessing SELDI-TOF mass spectrometry data
Source: BMC Bioinformatics. 2010 Oct 13;11:512. doi: 10.1186/1471-2105-11-512 (PMC2964685; doi:10.1186/1471-2105-11-512)
Supplement: Additional file 1 — Experiment and simulation settings. This file contains additional details about how simulations and experiments were carried out. [file 1471-2105-11-512-S1.PDF]

Supplementary Information
for

Emanuele II, VA, Gurbaxani, BM “Quadratic variance models for adaptively preprocessing SELDI-TOF mass spectrometry data” published in BMC Bioinformatics

1 Reproducible computational research

LibSELDI and the associated MATLAB scripts and data necessary to reproduce the figures and tables shown in the main text are available for download. For more information, contact the authors.

The files are available in zipped format (Windows users) or tarballs (Unix/Linux users). Once the files are downloaded, unzip all of the files in the same directory and read the the provided README.txt and LICENSE.txt files. The software is provided under version 3 of the GNU Public License.

2 Relevant SELDI PBS IIc settings

We summarize the machine settings used to generate the buffer+matrix only QA/QC spectra. We have selected what we believe to be the most pertinent factors affecting the results seen in Fig 1 and 2 of the main text. The lists in 2.2 and 2.3 are a summary of corresponding entries in the Ciphergen XML files produced. Additional parameters may be read directly out of the files. The QA/QC procedure used to produce the final BUFFER1 and BUFFER2 datasets is described in Section 2.1.

2.1 QA/QC (outlier removal)

We examine all spectra generated for BUFFER1 using the quantile spectrum approach described in the main text. For a fixed t, outlier points are detected as any points falling outside the the interval $[q_{25} - 1.5 \cdot IQR, q_{75} + 1.5 \cdot IQR]$, where q_{25}, q_{75}, and $IQR = q_{75} - q_{25}$ are the 25% quantile, 75% quantile, and inter-quartile range, respectively. This is done for all t. Any spectra containing one or more outlier points is declared an outlier and removed. This QA/QC procedure yields 183 high quality spectra for BUFFER1 and 114 high quality spectra for BUFFER2.

2.2 BUFFER1 settings (202 spectra, pre QA/QC)

- **spotProtocolInstructions:** Set high mass to 50000 Daltons, optimized from 3000 Daltons to 30000 Daltons. Set starting laser intensity to 185. Set starting detector sensitivity to 8. Focus by optimization center. Set Mass Deflector to 2000 Daltons. Set data acquisition method to Seldi Quantitation Set Seldi acquisition parameters 23. delta to 4. transients per to 12 ending position to 83. Set warming positions with 2 shots at intensity 195 and Do not include warming shots. Process sample.
- **ionFocusDelay:** 9.83e-007 s
- **deflectorMass:** 2000 Da
- **highMassCollected:** 50,000 Da
- **laserIntensityLow:** 185 (arbitrary units)
- **shotsFired:** 224
- **shotsKept:** 192
- **spotCorrectionEnabled:**
  - false (191 spectra)
  - true (11 spectra)

2.3 BUFFER2 settings (148 spectra, pre QA/QC)

- **spotProtocolInstructions:**
- Set high mass to 30000 Daltons, optimized from 3000 Daltons to 30000 Daltons. Set starting laser intensity to (multiple cases, see below). Set starting detector sensitivity to 7. Focus by optimization center. Set Mass Deflector to 2500 Daltons. Set data acquisition method to Seldi Quantitation Set Seldi acquisition parameters 20. delta to 4. transients per to 12 ending position to 80. Set warming positions with 2 shots at intensity 220 and Do not include warming shots. Process sample.
- **ionFocusDelay:** 9.83e-007 s
- **deflectorMass:** 2500 Da
- **highMassCollected:** 30,000 Da
- **laserIntensityLow:** (arbitrary units)
  - 185 (46 spectra), 190 (12 spectra), 195 (40 spectra), 200 (26 spectra), 210 (10 spectra), 215 (14 spectra)
- **shotsFired:** 224

- **shotsKept:** 192
- **spotCorrectionEnabled:**
  - true (148 spectra)
  - false (none)

3 MassSpecWavelet Code

Below we provide a code snippet to illustrate how MassSpecWavelet was used to calculate peak/protein predictions for each dataset over a wide range of snr settings in an efficient way. Note that MassSpecWavelet is implemented in the *R* computing language.

```
### ...,,, Calculate mean spectrum for directory
  print("Mean Spectrum Estimation\n")
  meanInt <- rowMeans(rawM$xtr)
  mzs <- rawM$mz

### ...,,, CWT
  print("CWT Calculation\n")
  wCoefs <- cwt( meanInt, scales = scales, wavelet = "mexh" )

  print("Analyzing CWT Result\n")
  wCoefs <- cbind( as.vector(meanInt), wCoefs)
  colnames(wCoefs) <- c(0,scales)
  localMax <- getLocalMaximumCWT(wCoefs)

### ...,,, Peak detection steps
  print("Looking for peaks/ridges\n")
  ridgeList <- getRidge(localMax)

  majorPeakInfo <- identifyMajorPeaks( meanInt, ridgeList, wCoefs,
                                      SNR.Th = 1, nearbyPeak=TRUE )

  # ...,,, Grab list of potential peaks and their SNRs
  potentialPeaks <- majorPeakInfo$potentialPeakIndex
  peakSNR <- majorPeakInfo$peakSNR[ names(potentialPeaks) ]
  uniqueSNR <- unique( peakSNR )
  sortedSNR <-sort( uniqueSNR, decreasing=TRUE )
  print("Done\n")
  # ... Create data structures for bookeeping
  peaks <- vector("list", length=length(sortedSNR) )
  attributes(peaks) <- list(names=rep("",length(sortedSNR)))
  parameters <- sortedSNR
```

```
# ...,,, Iterate over all SNRs and produce predictions.
for ( jsnr in 1:length(sortedSNR) ) {

  ## ... Get peaks with SNR at least this good
  thisSNR <- sortedSNR[ jsnr ]
  currentIndex <- peakSNR >= thisSNR
  currentPeaks <- potentialPeaks[ currentIndex ]

  peaks[[jsnr]] <- mzs[ currentPeaks ]

}
```
